# p53‐independent Noxa induction by cisplatin is regulated by ATF3/ATF4 in head and neck squamous cell carcinoma cells

**DOI:** 10.1002/1878-0261.12172

**Published:** 2018-04-17

**Authors:** Kanika Sharma, Thien‐Trang Vu, Wade Cook, Mitra Naseri, Kevin Zhan, Wataru Nakajima, Hisashi Harada

**Affiliations:** ^1^ Philips Institute for Oral Health Research School of Dentistry Massey Cancer Center Virginia Commonwealth University Richmond VA USA; ^2^ Department of Molecular Oncology Institute for Advanced Medical Sciences Nippon Medical School Kawasaki Japan

**Keywords:** apoptosis, ATF, cisplatin, ERK1, Noxa

## Abstract

The platinum‐based DNA damaging agent cisplatin is used as a standard therapy for locally advanced head and neck squamous cell carcinoma (HNSCC). However, the mechanisms underpinning the cytotoxic effects of this compound are not entirely elucidated. Cisplatin produces anticancer effects primarily via activation of the DNA damage response, followed by inducing BCL‐2 family dependent mitochondrial apoptosis. We have previously demonstrated that cisplatin induces the expression of proapoptotic BCL‐2 family protein, Noxa, that can bind to the prosurvival BCL‐2 family protein, MCL‐1, to inactivate its function and induce cell death. Here, we show that the upregulation of Noxa is critical for cisplatin‐induced apoptosis in p53‐null HNSCC cells. This induction is regulated at the transcriptional level. With a series of Noxa promoter‐luciferase reporter assays, we find that the CRE (cAMP response element) in the promoter is critical for the *Noxa* induction by cisplatin treatment. Among the CREB/ATF transcription factors, ATF3 and ATF4 are induced by cisplatin, and downregulation of ATF3 or ATF4 reduced cisplatin‐induced Noxa. ATF3 and ATF4 bind to and cooperatively activate the *Noxa* promoter. Furthermore, ERK1 is involved in cisplatin‐induced ATF4 and Noxa induction. In conclusion, ATF3 and ATF4 are important regulators that induce Noxa by cisplatin treatment in a p53‐independent manner.

**Abbreviations**
ATF: activating transcription factor
BCL‐2: B‐cell lymphoma‐2
BH3: BCL‐2 homology 3
CHOP: C/EBP‐homologous protein
CREB: CRE binding protein
CRE: cAMP response element
DMEM: Dulbecco's modified Eagle's medium
ERK: extracellular signal‐regulated kinase
GAPDH: Glyceraldehyde 3‐phosphate dehydrogenase
HNSCC: head and neck squamous cell carcinoma
MCL‐1: myeloid cell leukemia‐1
PARP: poly(ADP–ribose) polymerase

## 1 Introduction

Head and neck cancer is the sixth leading cancer worldwide. Head and neck squamous cell carcinoma (HNSCC) accounts for more than 90% of incident cases. Despite intense, multimodality treatment regimens for HNSCC including surgery, chemotherapy, and radiation, little progress has been made over the past 30 years in improving overall survival rates (Leemans *et al*., 2011; Rothenberg and Ellisen, 2012). Induction chemotherapy with platinum‐based compounds, taxanes, and 5‐fluorouracil is beneficial to head and neck cancer patients, but the prolonged use of chemotherapeutic drugs is limited by their toxicity and by the development of resistance (Schultz *et al*., 2010; Suh *et al*., 2014; Vermorken *et al*., 2007). Ultimately, cisplatin exerts anticancer effects via multiple mechanisms. Cisplatin is most prominent in the generation of DNA lesions, activation of DNA damage response, and the induction of p53 and BCL‐2 family dependent mitochondrial apoptosis. Tumor cell death induced by both conventional and targeted chemotherapy is often mediated by the BCL‐2 family dependent mitochondrial apoptotic pathway (Adams and Cory, 2007; Chipuk *et al*., 2010; Youle and Strasser, 2008). However, initiators of this apoptotic pathway, such as p53, are frequently mutated or deleted in HNSCC rendering the disease refractory to treatment (Poeta *et al*., 2007; Sano *et al*., 2011). The development of chemoresistance is often associated with cisplatin treatment, which leads to therapeutic failure. However, the mechanisms of the resistance by cisplatin remain unclear.

The BCL‐2 family consists of three main groups: prosurvival (e.g., BCL‐2, MCL‐1, BCL‐X_L_), multidomain prodeath (e.g., BAX, BAK), and BH3‐only prodeath (e.g., BAD, BID, BIM, Noxa). BH3‐only proteins cause cytochrome c release from the mitochondria by activating BAX and/or BAK, while the prosurvival BCL‐2 family of proteins prevents this process (Adams and Cory, 2007; Chipuk *et al*., 2010; Youle and Strasser, 2008). In the case of cisplatin‐induced apoptosis, a BH3‐only protein, Noxa, specifically binds to and recruits the prosurvival MCL‐1 from the cytosol to the mitochondria to inactivate the function of MCL‐1. Translocation of MCL‐1 initiates its phosphorylation and subsequent ubiquitination, which triggers proteasome‐mediated degradation (Nakajima *et al*., 2016). It has been demonstrated that the transcription of Noxa is induced by cisplatin, which is both p53‐dependent and p53‐independent mechanisms (Gutekunst *et al*., 2013; Sheridan *et al*., 2010; Zhu *et al*., 2013). Although, Noxa was originally identified as a p53 target gene, the p53‐independent mechanisms of the transcription of Noxa are not entirely elucidated. To investigate the mechanisms of cisplatin‐induced, p53‐independent apoptosis, we used HN8 and HN12 HNSCC cell lines in which p53 was inactive (Yeudall *et al*., 1997). Cisplatin treatment on these cells triggers the induction of Noxa and apoptosis. We found that the upregulation of Noxa was critical for cisplatin‐induced apoptosis in a p53‐independent manner. Therefore, we focused on the p53‐independent mechanism of Noxa induction by cisplatin treatment. We identified that transcription factors, ATF3 and ATF4, contribute to regulate *Noxa* mRNA induction by cisplatin treatment through CRE on the promoter. We further analyzed the signaling pathways to regulate ATF3 and ATF4 induction by cisplatin.

## 2 Materials and methods

### 2.1 Cell lines and cell culture

HN8 and HN12 cells were kindly provided by W. Andrew Yeudall (Augusta University). Cells were cultured in Dulbecco's modified Eagle's medium (DMEM; Life Technologies, Grand Island, NY, USA) supplemented with 10% heat‐inactivated fetal bovine serum (FBS) and 100 μg·mL^−1^ penicillin G/streptomycin at 37 °C in a humidified, 5% CO_2_ incubator.

### 2.2 Lentivirus production

The lentiviral short‐hairpin RNA (shRNA)‐expressing constructs were purchased from Sigma‐Aldrich (St. Louis, MO, USA). The target sequences for each shRNA are the following: Noxa 2: 5′‐CTTCCGGCAGAAACTTCTGAA‐3′, Noxa 4: 5′‐TGGAAGTCGAGTGTGCTACTC‐3′, ATF3‐1: 5′‐GCTGAACTGAAGGCTCAGATT‐3′, ATF3‐2: 5′‐CTTCATCGGCCCACGTGTATT‐3′, ATF4‐1: 5′‐GCCTAGGTCTCTTAGATGATT‐3′, ATF4‐2: 5′‐GCCAAGCACTTCAAACCTCAT‐3′, ERK1: 5′‐CCTGAATTGTATCATCAACAT‐3′, ERK2‐1: 5′‐CAAAGTTCGAGTAGCTATCAA‐3′, ERK2‐2: 5′‐TATCCATTCAGCTAACGTTCT‐3′, CREB: 5′‐ACAGCACCCACTAGCACTATT‐3′. The constructs were transfected into 293T packaging cells along with the packaging plasmids using EndoFectin Lenti (GeneCopoeia, Rockville, MD, USA) and the lentivirus‐containing supernatants were used to transduce the cells.

### 2.3 Luciferase assay

The sequences of p53 and CRE mutants on the *Noxa* promoter are the following: p53: 5′‐GAGAGTTTCCGGGAAGTTCGCG‐3′, CRE: 5′‐CTAAAAAA‐3′. Each promoter construct (−198 to +157 from the transcription start site) was cloned into KpnI‐BglII sites in PGV‐B2 (Toyo B‐Net, Tokyo, Japan). The ATF3 and ATF4 expression vectors were purchased from Addgene (Cambridge, MA, USA) (Wang *et al*., 2010). HN8 or HN12 cells (1 × 10^5^ cells/12 well) were transfected with 0.5 μg of reporter plasmids, 0.4 μg of an ATF3 expression plasmid, an ATF4 expression plasmid (wild‐type or deletion mutants) or an empty vector, and 0.1 μg pRL‐SV40 *Renilla* luciferase plasmid (Promega, Madison, WI, USA) using EndoFectin Max (GeneCopoeia). Luciferase activity was measured using the Dual‐Luciferase Reporter System (Promega) and normalized to the *Renilla* luciferase activity expressed by pRL‐SV40.

### 2.4 Chemicals and antibodies

Cisplatin and SP600125 were purchased from ApexBio (Houston, TX, USA). SB203580 and PD184352 were purchased from LC Laboratories (Woburn, MA, USA). Cisplatin was dissolved in PBS and other reagents were dissolved in dimethyl sulfoxide (Hall *et al*., 2014). The following antibodies were used: cleaved‐PARP (D64E10), ATF4, CHOP and GAPDH from Cell Signaling Technology (Danvers, MA, USA); Noxa (114C307.1) from Thermo Fisher Scientific (Waltham, MA, USA); MCL‐1 from Enzo Life Sciences (Farmingdale, NY, USA); ATF3, ATF4, pERK, ERK1, and ERK2 from Santa Cruz Biotechnology (Santa Cruz, CA, USA); ATF4 from GeneTex (Irvine, CA, USA).

### 2.5 Western blot analyses

Whole cell lysates were prepared with CHAPS lysis buffer [20 mm Tris (pH 7.4), 137 mm NaCl, 1 mm DTT, 1% CHAPS, a protease inhibitor cocktail, and phosphatase inhibitor cocktails (Sigma‐Aldrich)]. Equal amounts of proteins were loaded on a SDS acrylamide gel, transferred to a nitrocellulose membrane, and analyzed by immunoblotting with ECL2 (Thermo Scientific, Rockford, IL, USA).

### 2.6 Cell viability assay

Cell death was quantified by Annexin V‐FITC (BD Biosciences, San Jose, CA, USA)‐propidium iodide (Sigma‐Aldrich) staining according to the manufacturer's protocol, followed by flow cytometric analysis using FACScan (BD Biosciences).

### 2.7 Quantitative real‐time PCR

Quantitative real‐time PCR analysis was performed as previously described. The primer and probe sets (GAPDH, Hs99999905_m1; Noxa, Hs00560402_m1) were purchased from Applied Biosystems (Carlsbad, CA, USA). Data were analyzed as mRNA expression levels relative to GAPDH according to the manufacturer's protocol.

### 2.8 Chromatin immunoprecipitation assay

The ChIP assay was performed using the kit (Cell Signaling Technology) according to the manufacture's instruction. To amplify the DNA, the following primers were used: Forward: 5′‐CCTACGTCACCAGGGAAGTT‐3′, Reverse: 5′‐GATGCTGGGATCGGGTGT‐3′.

### 2.9 Statistical analysis

Values represent the means ± SD for triplicates. The significance of differences between the experimental variables was determined using the Student's *t*‐test. Values were considered statistically significant at *P* < 0.05.

## 3 Results

### 3.1 Cisplatin‐induced apoptosis is Noxa‐dependent in HNSCC cells

**Figure 1:**
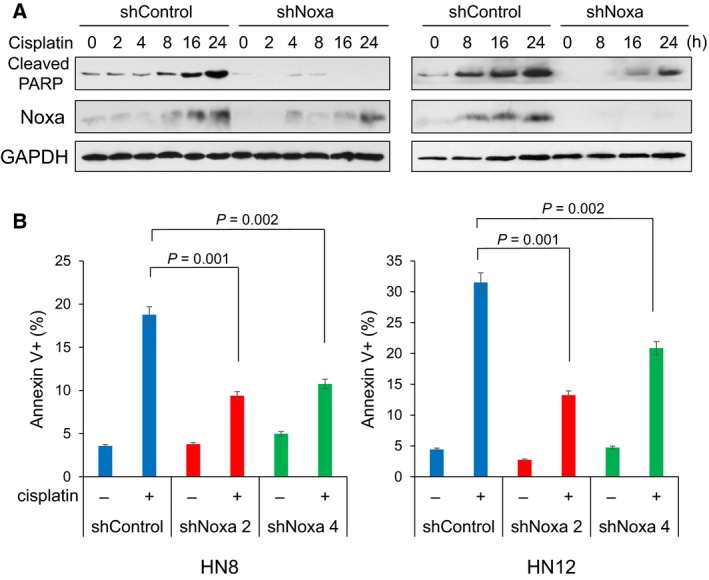
Noxa contributes to cisplatin‐induced apoptosis in a p53‐independent manner. (A) p53‐inactive HN8 and HN12 HNSCC cells were infected with lentiviruses encoding shRNA for nontargeting control or Noxa (shNoxa2). Cells were treated with cisplatin (50 μm for HN8 or 25 μm for HN12) with the indicated periods and equal amounts of the total extracts were used for immunoblot analysis with the indicated antibodies. (B) The cells in (A) were treated with cisplatin for 24 h and cell death was determined by Annexin V‐propidium iodide staining followed by FACS analyses. Another shNoxa construct, shNoxa4 was also introduced in each cell line, which was assayed similarly as shNoxa2. Values represent the mean ± SD of triplicates. The results are representative of at least two independent experiments and are reproducible.

It has been demonstrated that treatment with DNA‐damaging agents, such as cisplatin, induces Noxa expression and apoptosis in a variety of cell lines (Gutekunst *et al*., 2013; Lin *et al*., 2012; Nakajima *et al*., 2016; Sheridan *et al*., 2010; Zhu *et al*., 2013). To explore the significance of Noxa in cisplatin‐induced cell death in HNSCC, we first stably expressed the shRNA for Noxa or scrambled shRNA as control in HN8 (*p53* deleted) and HN12 (p53 truncated and inactivated) cells (p53 expression is shown in Fig. S1) and then treated with cisplatin with the IC_50_ concentrations (50 μm for HN8 or 25 μm for HN12). In the control cells, Noxa and cleaved‐PARP (indicative of apoptosis) were induced starting at 8 h (Fig. 1A). Downregulation of Noxa resulted in reduction of cisplatin‐induced apoptosis, as judged by PARP cleavage and Annexin V staining (Fig. 1 and Fig. S2). These results suggest that Noxa is required for cisplatin‐induced apoptosis in HNSCC cells.

### 3.2 The CRE on the *Noxa* promoter is essential for cisplatin‐mediated Noxa induction independent of p53

**Figure 2:**
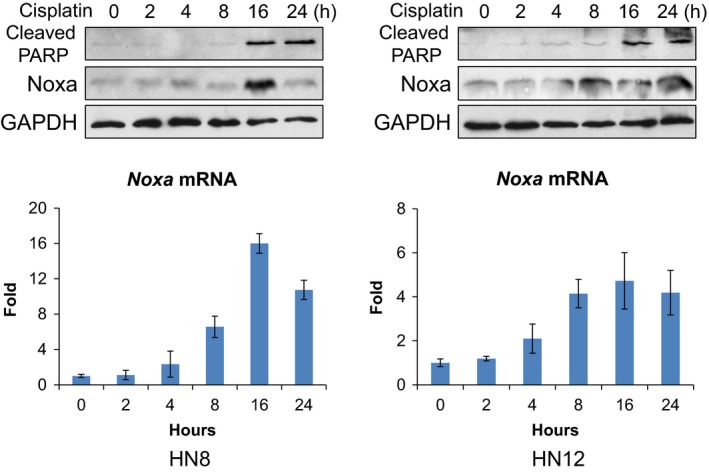
*Noxa* mRNA is induced by cisplatin in p53‐null HNSCC cells. Following cisplatin treatment (50 μm for HN8 or 25 μm for HN12), Noxa protein and mRNA expression at each time point were determined by western blots (upper panel) and quantitative real‐time PCR (lower panel), respectively. Values represent the mean ± SD of triplicates.

Although originally identified as a p53 target gene, Noxa is induced and required for cisplatin‐induced cell death even in p53‐null cell lines, such as HN8 and HN12 (Fig. 1). Thus, we explored the mechanisms of p53‐independent Noxa induction. In both HN8 and HN12 cells, gradual increases of *Noxa* mRNA were observed from 0 to 16 h, which correlated with Noxa protein induction (Fig. 2). This result suggests that the increase in Noxa expression is controlled by the mRNA level. Thus, our focus shifted to understanding the transcriptional regulation of *Noxa*.

**Figure 3:**
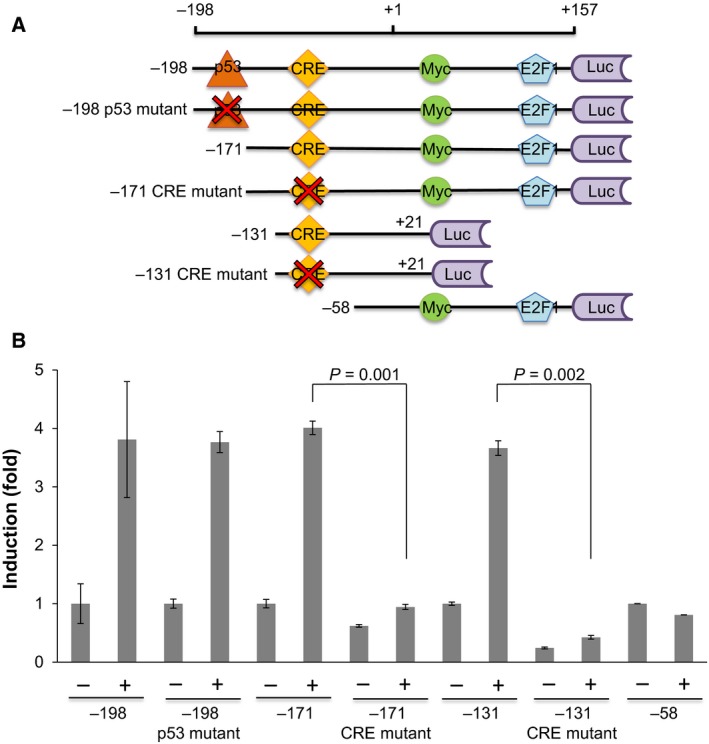
A CRE on the Noxa promoter is critical for cisplatin‐induced Noxa expression. (A) Schematic representation of the promoter region of *Noxa*. The mutations and deletions of the *Noxa* promoter were used for the promoter‐luciferase reporter fusion gene constructs. (B) A series of luciferase constructs shown in (A) were transfected in HN8 cells. On the next day, cells were treated with 50 μm of cisplatin for 16 h. Values represent the mean ± SD of triplicates. The results are representative of at least two independent experiments and are reproducible.

We next determined the promoter regions required for *Noxa* induction using a series of *Noxa* promoter‐luciferase constructs (Fig. 3A). In *p53*‐deleted HN8 cells, there was ~ 4‐fold induction with the −198 construct (−198 to +157 from the transcription start site). When the p53 binding site was mutated (−198 p53 mutant), similar levels of induction was still observed with cisplatin treatment. Consistently, there was ~ 4‐fold induction in the −171 construct in which the p53 binding site was deleted (Fig. 3B). The results from these constructs confirm that *Noxa* can be induced by cisplatin in a p53‐independent manner. When the CRE was mutated (−171 CRE mutant), cisplatin‐mediated Noxa induction was not detected (Fig. 3B). We further generated −131 to +21 construct and its CRE mutant (Fig. 3A). The intact −131 construct showed a ~ 4‐fold induction, whereas the −131 CRE mutant construct did not show the induction by cisplatin treatment (Fig. 3B). There was no induction with the −58 construct in which both the p53 binding site and CRE were deleted, but Myc and E2F binding sites were retained (Fig. 3B). Similar results were obtained using HN12 cells, confirming the requirement of CRE for cisplatin‐mediated Noxa induction (Fig. S3). Taken together, these data indicate that the CRE is critical for cisplatin‐mediated *Noxa* induction independent of the p53 binding site.

### 3.3 ATF3 and ATF4 regulate cisplatin‐mediated Noxa induction through binding to the CRE on the *Noxa* promoter

**Figure 4:**
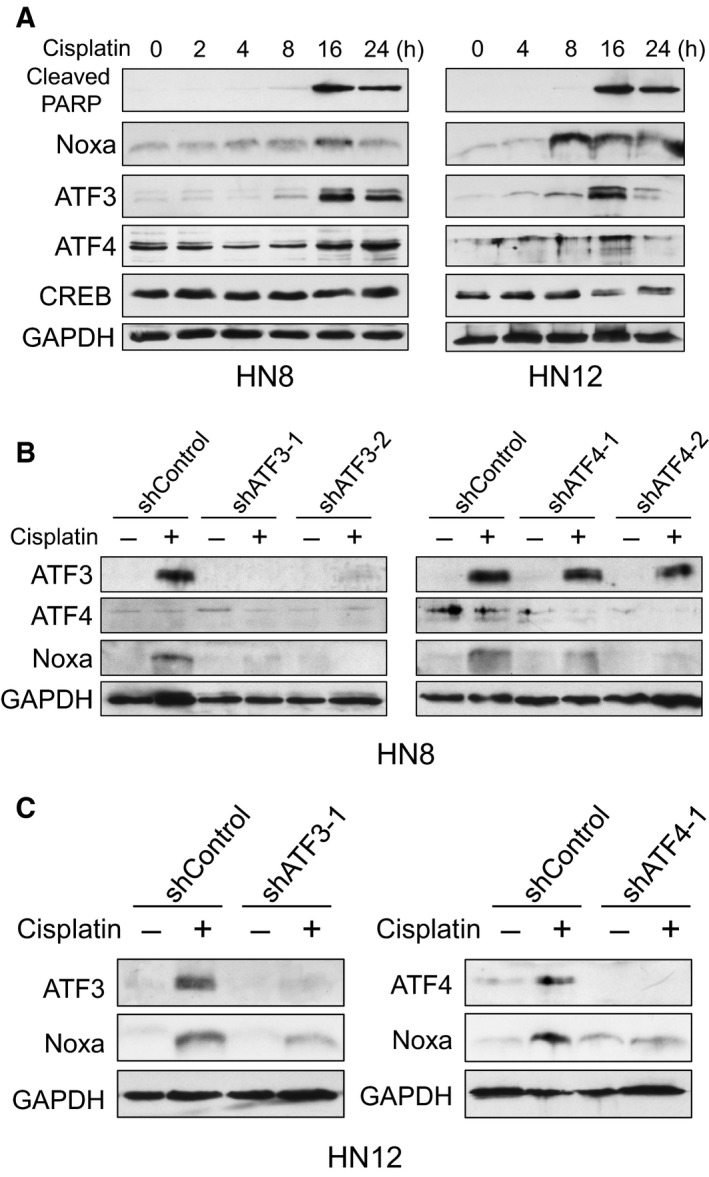
ATF3 and ATF4 contribute to Noxa induction by cisplatin treatment. (A) HN8 and HN12 cells were treated with cisplatin (50 μm for HN8 or 25 μm for HN12) for the indicated times. Equal amounts of the total extracts were used for immunoblot analysis with the indicated antibodies. (B, C) HN8 (B) or HN12 (C) cells were infected with lentiviruses encoding shRNA for nontargeting control, ATF3, or ATF4. Cells were treated with cisplatin for 16 h and equal amounts of the total extracts were used for immunoblot analysis with the indicated antibodies.

It has been demonstrated that CRE is regulated by the ATF/CREB family transcription factors such as ATF3, ATF4, and CREB (Ameri and Harris, 2008; Servillo *et al*., 2002). We first determined the expression of these transcription factors after cisplatin treatment in HN8 and HN12 cells. ATF3 showed the highest expression at 16 h with cisplatin treatment when Noxa and cleaved‐PARP were at the peak (Fig. 4A). ATF4 was slightly induced at 16 h. In contrast, CREB showed relatively constant expression (Fig. 4A).

We then downregulated each transcription factor by specific shRNAs to examine which transcription factor was contributing to *Noxa* induction. The induction of Noxa was strongly reduced by shRNAs for ATF3 and ATF4 compared to shControl in both HN8 and HN12 cells (Fig. 4B,C). In contrast, downregulation of CREB by shRNA did not affect Noxa induction (Fig. S4). These results suggest that ATF3 and ATF4 control cisplatin‐mediated Noxa induction independent of p53.

**Figure 5:**
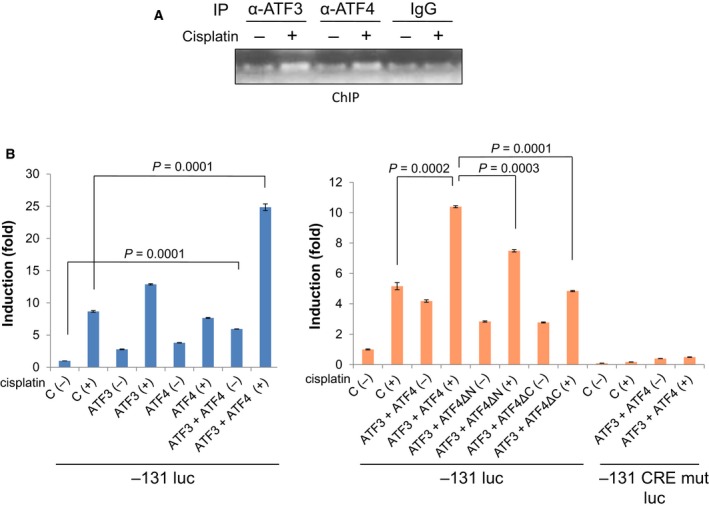
ATF3 and ATF4 bind to and activate the Noxa promoter through the CRE. (A) HN8 cells were treated with 50 μm of cisplatin for 8 h. Chromatin immunoprecipitation was performed with the indicated antibodies and DNA was analyzed by PCR using primers corresponding to −67 to +60 on the *Noxa* promoter. (B) HN12 cells were cotransfected with the −131 luc reporter construct and an ATF3 or ATF4 expression plasmid for 24 h. On the next day, cells were treated with 25 μm of cisplatin for 16 h. Values represent the mean ± SD of triplicates. The results are representative of at least two independent experiments and are reproducible.

Next, we examined whether endogenous levels of ATF3 and ATF4 bind to CRE in the Noxa promoter. We performed a ChIP assay using ATF3 or ATF4 specific antibodies and the primers that included CRE. Eight hours after cisplatin treatment, the binding of ATF3 and ATF4 to the DNA element containing CRE was clearly increased (Fig. 5A), suggesting that these transcription factors indeed bind to the CRE.

Furthermore, in order to address whether ATF3 and ATF4 cooperatively activate the *Noxa* promoter, we transfected ATF3 and/or ATF4 expression vectors together with the −131 *Noxa* promoter‐luciferase construct that contains the CRE for induction (Fig. 3). ATF3 or ATF4 alone slightly activated the *Noxa* promoter, which was enhanced by cotransfection of ATF3 and ATF4 [cisplatin (–) in Fig. 5B and Fig. S5, left panels]. All these activities were augmented by cisplatin treatment. It has been demonstrated that ATF3 and ATF4 physically associate and activate the *Noxa* promoter in mantle cell lymphoma cells (Wang *et al*., 2010). This paper showed that the N‐terminal domain of ATF4 was dispensable for interaction with ATF3, and the C‐terminal DNA binding domain was required for interaction with ATF3. When deletion mutants of each domain of ATF4 were transfected, the activation of *Noxa* promoter was less compared to ATF4 wild‐type, but higher than the vector‐only control particularly without cisplatin (Fig. 5B and Fig. S5, right panels), suggesting that both DNA binding of ATF4 and interaction with ATF3 and ATF4 are required for cooperative activation of the *Noxa* promoter. The activation mediated by ATF3 and ATF4 was completely abrogated with a CRE mutant of the *Noxa* promoter. These results altogether suggest that ATF3 and ATF4 bind to the CRE of the *Noxa* promoter to cooperatively induce the *Noxa* expression.

### 3.4 ATF4 activation is regulated through ERK1 by cisplatin treatment

**Figure 6:**
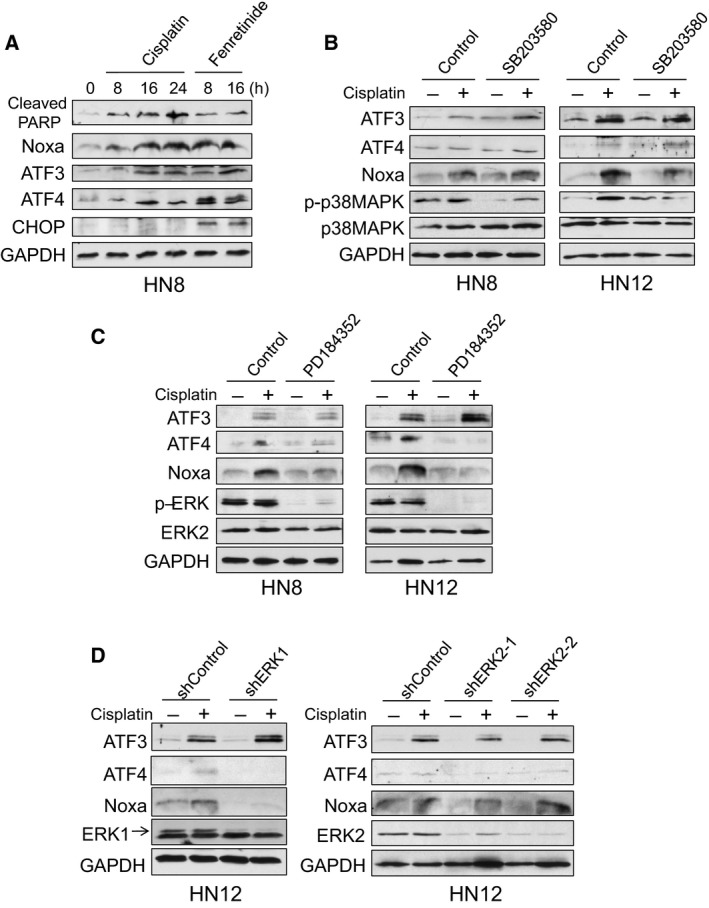
Cisplatin‐induced Noxa is regulated by ERK1 through ATF4 induction. (A) HN8 cells were treated with cisplatin (50 μm) or fenretinide (10 μm) for the indicated times. Equal amounts of the total extracts were used for immunoblot analysis with the indicated antibodies. (B) HN8 and HN12 cells were pretreated with a p38‐MAPK inhibitor, SB203580 (5 μm) for 30 min, and then treated with cisplatin for 16 h (50 μm for HN8 or 25 μm for HN12). Equal amounts of the total extracts were used for immunoblot analysis with the indicated antibodies. (C) HN8 and HN12 cells were pretreated with a MEK inhibitor, PD184352 (5 μm) for 30 min, and then treated with cisplatin for 16 h. Equal amounts of the total extracts were used for immunoblot analysis with the indicated antibodies. (D) HN12 cells were infected with lentiviruses encoding shRNA for nontargeting control, ERK1, or ERK2. Cells were treated with 25 μm of cisplatin for 16 h and equal amounts of the total extracts were used for immunoblot analysis with the indicated antibodies.

We next addressed the signaling pathways for the induction of ATF3 and ATF4 in cisplatin treatment. Noxa is known to be induced by ATFs with endoplasmic reticulum (ER) stress inducers, such as fenretinide and Eeyarestatin I (Qing *et al*., 2012; Wang *et al*., 2010). Fenretinide induced ATF3, ATF4, and Noxa similarly as cisplatin treatment. However, cisplatin did not induce an ER stress marker, CHOP, which was clearly induced by fenretinide (Fig. 6A), suggesting that ER stress was not induced by cisplatin treatment.

It has been shown that the MEK‐ERK or p38‐MAPK pathways are involved in Noxa induction in cisplatin treatment (Sheridan *et al*., 2010; Zhu *et al*., 2013). Thus, we used a MEK inhibitor, PD184352, and a p38‐MAPK inhibitor, SB203580, to examine the effect on cisplatin‐induced Noxa expression. Pretreatment with SB203580 affected neither Noxa nor ATF3/ATF4 induction by cisplatin treatment (Fig. 6B). In contrast, pretreatment with PD184352 strongly inhibited Noxa induction in both HN8 and HN12 cells (Fig. 6C). The induction of ATF4, but not ATF3, was also strongly inhibited. It has been reported that cisplatin‐induced JNK activation is a key regulator of ATF3 induction in nonsmall cell lung carcinoma cells (Bar *et al*., 2016). Thus, we also tested the involvement of JNK for Noxa induction using a JNK inhibitor, SP600125. The inhibition of JNK affected neither ATF3 nor Noxa induction in HNSCC cells (Fig. S6). These results suggest that the MEK‐ERK pathway regulates Noxa induction through ATF4 activation independent of p53.

The MEK inhibitor, PD184352, equally inactivates ERK1 and ERK2 (pERK in Fig. 6C). ERK1 and ERK2 are thought as redundant isoforms due to high sequence homology. However, these proteins play a specific role in certain circumstances (Busca *et al*., 2016; Mehdizadeh *et al*., 2016). Thus, we performed specific downregulation by each shRNA to examine which ERK regulates ATF4 and Noxa by cisplatin. Downregulation of ERK1 clearly inhibited Noxa induction as well as ATF4 (Fig. 6D, Left panel). In contrast, downregulation of ERK2 did not reduce cisplatin‐induced ATF3, ATF4 or Noxa levels (Fig. 6D, Right panel). Taken together, ERK1 predominantly regulates cisplatin‐induced ATF4 and Noxa induction.

## 4 Discussion

DNA damaging agents, such as cisplatin, have been commonly used for chemotherapy in solid tumors for decades, but the molecular mechanisms of the action are still obscure. We and others have shown that a proapoptotic BCL‐2 family protein, Noxa plays a key role to induce cell death by cisplatin (Gutekunst *et al*., 2013; Lin *et al*., 2012; Nakajima *et al*., 2016; Sheridan *et al*., 2010; Zhu *et al*., 2013). Although Noxa was originally identified as a p53‐target gene, many cancers have p53 inactivation by deletion or mutations. Thus, it is important to identify p53‐independent regulatory mechanisms of Noxa in cisplatin treatment for overcoming the resistance. Using p53‐inactive HNSCC cells, we find that cisplatin‐induced Noxa is mainly regulated at the transcriptional level. Furthermore, the promoter analyses identified that CRE is crucial for the *Noxa* induction and transcription factors, ATF3 and ATF4 play an important role in this regulation. It has been shown that Noxa is regulated by a variety of signaling pathways through ATF3/ATF4 (Bagheri‐Yarmand *et al*., 2015; Qing *et al*., 2012; Wang *et al*., 2010; Yan *et al*., 2014). Thus, this study adds another pathway to induce Noxa through ATF3/ATF4. Although downregulation of Noxa by shRNA strongly inhibited cisplatin‐induced apoptosis, residual cell death activity was observed (Fig. 1), suggesting the involvement of other BH3‐only proteins. It has been shown that BIM is upregulated upon DNA‐damaging agent in a p53‐independent manner (Delbridge *et al*., 2016; Happo *et al*., 2010). Further studies are needed to clarify this point.

ATF3 and ATF4 are often activated by ER stress. However, cisplatin did not activate CHOP, an ER stress marker. In contrast, fenretinide induced ER stress, and ATF3, ATF4, and Noxa were induced as similar as those by cisplatin treatment (Fig. 6A). This result suggests that cisplatin and fenretinide induce Noxa mainly through ATF3/ATF4 in a p53‐independent manner, but the regulatory signaling pathways are distinct. A MEK inhibitor, PD184352 specifically inhibited cisplatin‐induced ATF4 followed by Noxa (Fig. 6C). Furthermore, ERK1, but not ERK2, plays a predominant role for this induction (Fig. 6D). The specific role of ERK1 in cisplatin induction has also been shown in hepatocellular carcinoma cells (Guegan *et al*., 2013). These results suggest that combination treatment with cisplatin and MEK inhibitors may not be effective for cancer therapies, since this combination may not synergize, but antagonize the effect of treatment. Cisplatin also activates p38‐MAPK (Zhu *et al*., 2013) or JNK (Bar *et al*., 2016), but treatment with neither a p38‐MAPK inhibitor SB203580 nor a JNK inhibitor SP600125 affected cisplatin‐induced Noxa or ATF3/ATF4 (Fig. 6B and Fig. S6), suggesting that p38‐MAPK and JNK play alternative roles in cisplatin‐induced apoptosis. Thus, it is still unclear how ATF3 is induced by cisplatin. Once the signaling pathways of ATF3 activation become elucidated, the activators of these pathways could be alternative strategies for targeting Noxa to efficiently induce cell death for cancer treatment.

Our results indicate that Noxa can be efficiently induced by not only cisplatin, but also by an ER stress inducer, fenretinide, through ATF3/ATF4 activation (Fig. 6A). The induction of Noxa mainly and solely targets MCL‐1 to inactivate its function. Thus, combining the two treatments to inhibit MCL‐1 together with BCL‐2/BCL‐X_L_ should effectively induce apoptosis in cancer cells. Indeed, when HN8 and HN12 cells were treated with fenretinide and ABT‐263 (navitoclax), we observed synergistic induction of cell death (unpublished results). It has been demonstrated that combination of fenretinide and ABT‐737, a preclinical compound of ABT‐263, shows enhanced apoptosis in neuroblastoma, melanoma, and B‐cell chronic lymphocytic leukemia models (Bruno *et al*., 2012; Fang *et al*., 2011; Mukherjee *et al*., 2015). Since fenretinide and navitoclax are used in clinical trials in a variety of cancers, this combination might become an alternative strategy to treat HNSCC.

## 5 Conclusions

**Figure 7:**
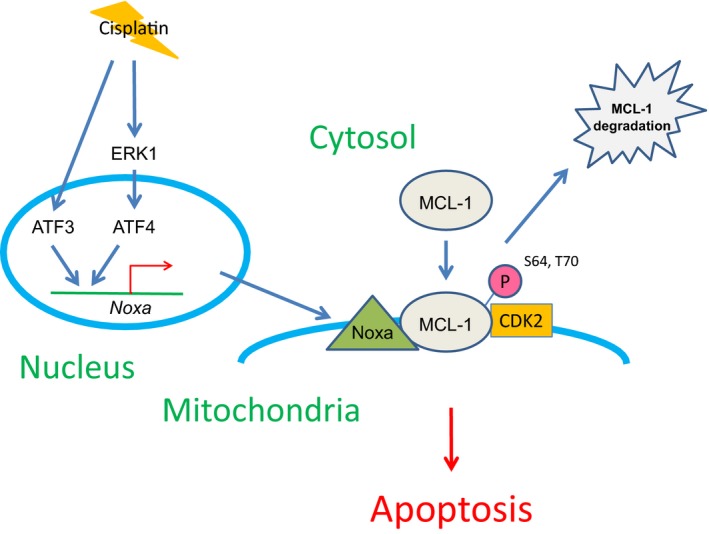
A schematic representation of the signaling pathway from cisplatin treatment to the induction of apoptosis.

We have previously demonstrated that Noxa‐mediated MCL‐1 phosphorylation followed by MCL‐1 degradation is critical for apoptosis induced by DNA damaging agents through regulation of the Noxa/MCL‐1/CDK2 complex (Nakajima *et al*., 2016). In the current study, Noxa induction is mediated by ATF3/ATF4 transcription factors, which are regulated specifically by ERK1 in the upstream signaling pathway via cisplatin treatment. Taken together, we propose one of the signaling pathways that cisplatin takes to induce apoptosis, as shown in Fig. 7. We expect some of these proposed molecules in the figure could be used as biomarkers and targets to enhance the cisplatin sensitivity to overcome resistance.

## Author contributions

KS and HH conceived and designed the experiments. WN provided a series of luciferase constructs. KS, T‐TV, WC, MN, and KZ carried out the experiments. KS and HH wrote the manuscript.

## Supporting information


**Fig. S1.** The expression of p53 in HNSCC cell lines.
**Fig. S2.** Noxa contributes to cisplatin‐induced apoptosis.
**Fig. S3.** A CRE on the Noxa promoter is critical for cisplatin‐induced Noxa expression.
**Fig. S4.** The involvement of CREB in cisplatin‐mediated Noxa induction.
**Fig. S5.** The expression of ATF3 and ATF4 transfected in HN12 cells.
**Fig. S6.** The involvement of JNK in cisplatin‐mediated Noxa induction.
