# Supplementary figures and images for: p53‐independent Noxa induction by cisplatin is regulated by ATF3/ATF4 in head and neck squamous cell carcinoma cells
Source: Mol Oncol. 2018 Apr 17;12(6):788–98. doi: 10.1002/1878-0261.12172 (PMC5983129; doi:10.1002/1878-0261.12172)

Figure S1

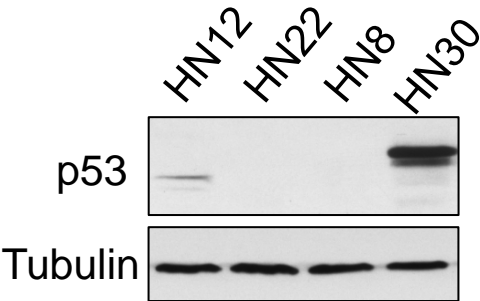

## Figure S2

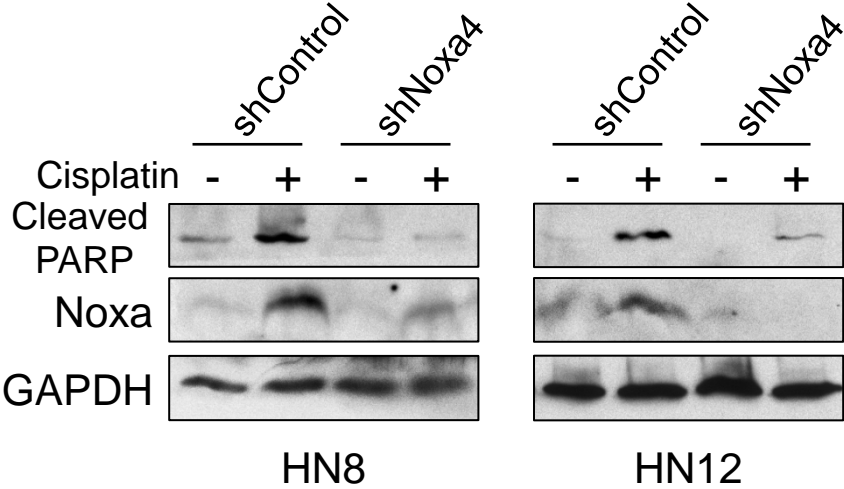

Figure S3

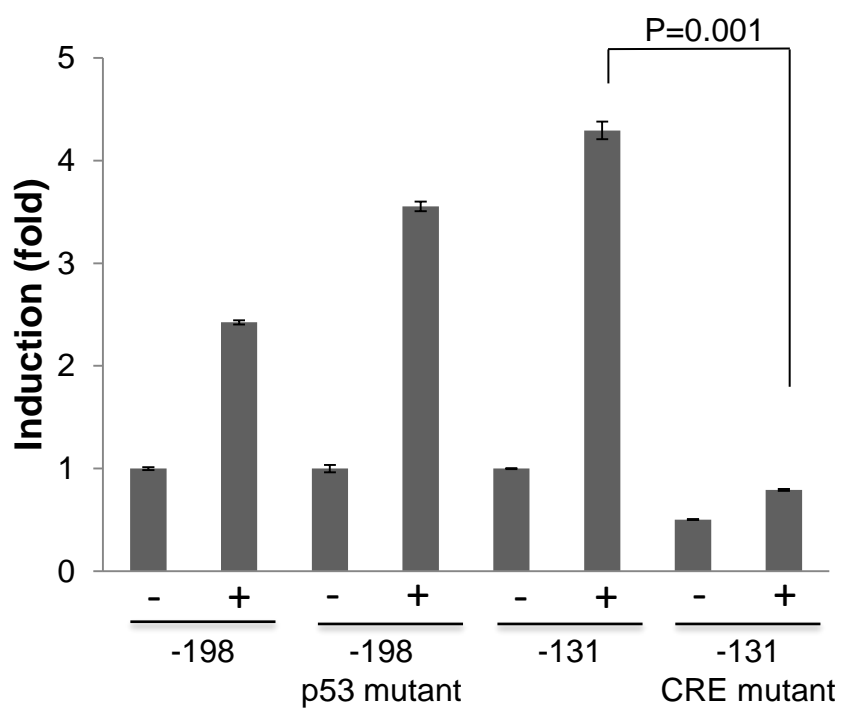

Figure S4

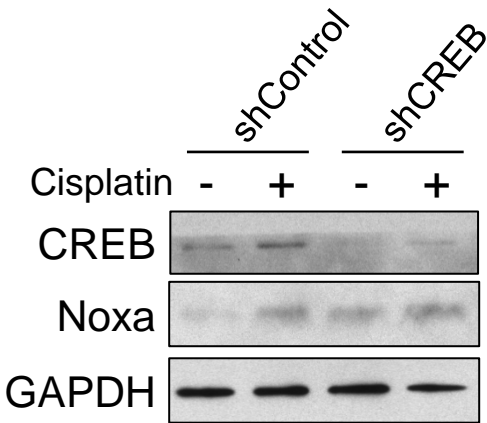

Figure S5

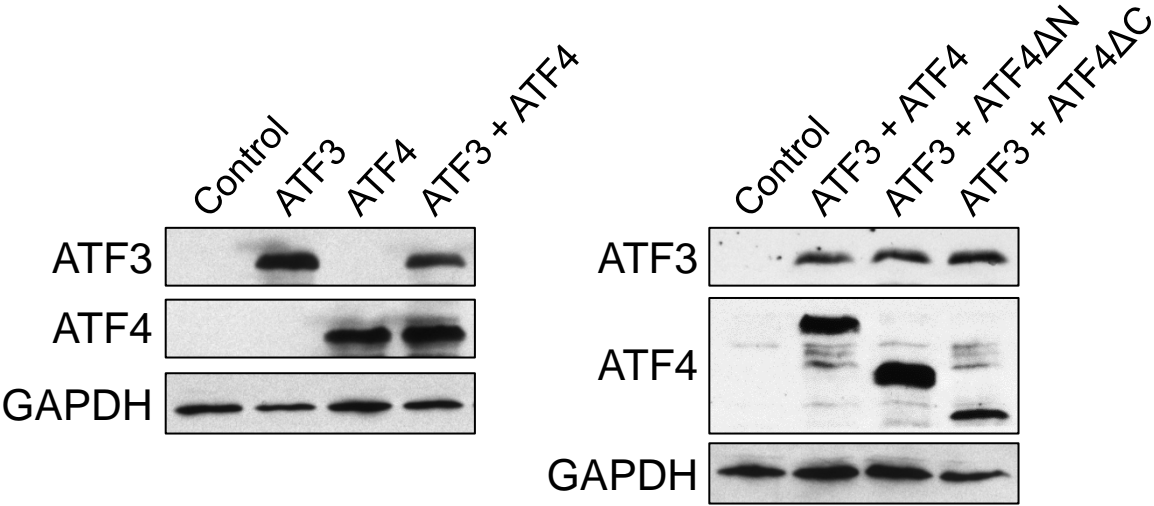

## Figure S6

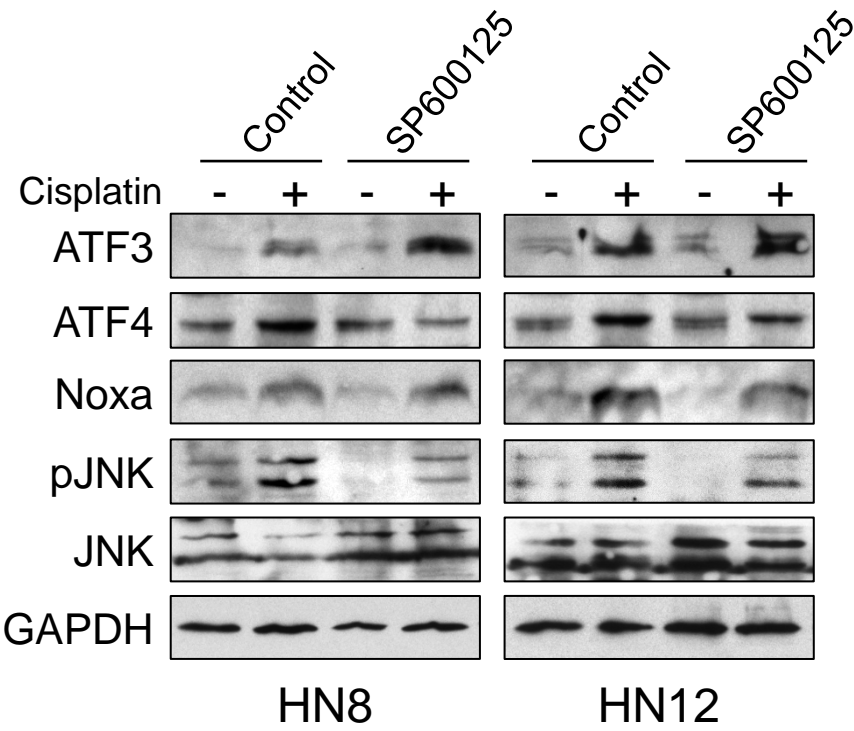

Supplement: Supplementary file 1 — Fig. S1. The expression of p53 in HNSCC cell lines. Fig. S2. Noxa contributes to cisplatin‐induced apoptosis. Fig. S3. A CRE on the Noxa promoter is critical for cisplatin‐induced Noxa expression. Fig. S4. The involvement of CREB in cisplatin‐mediated Noxa induction. Fig. S5. The expression of ATF3 and ATF4 transfected in HN12 cells. Fig. S6. The involvement of JNK in cisplatin‐mediated Noxa induction. [file MOL2-12-788-s001.pdf]
